# Factors affecting behaviors during complementary feeding in infants and children aged 6–24 months

**DOI:** 10.1371/journal.pone.0314694

**Published:** 2025-01-03

**Authors:** Yagmur Demirel Ozbek, Isa Celik, Aysenur Sahin Bilgin

**Affiliations:** 1 Department of Nutrition and Dietetics, Faculty of Health Sciences, Recep Tayyip Erdoğan University, Rize, Turkey; 2 Department of Pediatric Nursing, Faculty of Health Sciences, Recep Tayyip Erdogan University, Rize, Turkey; 3 Vitamince Nutrition Counseling, Maltepe/Istanbul, Turkey; Norwegian Institute of Public Health: Folkehelseinstituttet, NORWAY

## Abstract

The process that begins around the 6th month of life and continues until the 24th month is called the complementary feeding period. During this period, infants and children start receiving foods that complement breast milk or formula for the first time. The psychosocial factors the infants and children encounter during this period may affect their growth and health in later life. This cross-sectional and descriptive study aimed to examine the factors influencing behaviors of infants and children during complementary feeding. The study sample included 345 mothers with infants and children aged 6–24 months. The research data were collected using two forms and one scale. The first form contained questions about the mothers’ sociodemographic characteristics, sources of support in childcare, and information sources related to complementary feeding. The second form contained questions about the sociodemographic characteristics of infants and children, the presence of allergies, breastfeeding, and feeding status. The scale used was the validated Behaviors of Transition to Complementary Feeding Scale. The effect of independent variables on behavior of infants and children during complementary feeding was examined using multiple linear regression analysis. Infants/children older than 12 months exhibited more negative behaviors during complementary feeding compared with those aged 6–12 months. The study also found that being the first infant/child in the family had a negative impact on behaviors during complementary feeding. Infants and children currently receiving only complementary feeding displayed more positive behaviors during the complementary feeding process. Paternal support in childcare positively influenced behaviors during this period. In conclusion, complementary feeding is a multifaceted process influenced by various factors, including the infant’s and child’s age, family dynamics, and parental support. Strategies to support mothers, involve fathers, and provide reliable information can facilitate a smoother process of complementary feeding and promote healthier feeding behaviors in infants and children. Descriptive, interventional, qualitative, and mixed-methods studies are required to analyze these factors in detail and improve the complementary feeding process.

## Introduction

The first thousand days of life refer to the period from the beginning of pregnancy until the second birthday of the infant and child [1]. An increasing number of studies have demonstrated the vital role of this period in the physiology, function, performance, and health status of individuals [2, 3]. They have also emphasized that nutrition of infants and children in the first thousand days may impact the risk of developing noninfectious diseases such as obesity, arterial hypertension, cardiovascular diseases, and type 2 diabetes mellitus in adulthood [4–6].

Breastfeeding can provide adequate nutrition for most infants and children during the first 6 months of life [7]. The American Academy of Pediatrics recommends that infants should be fed exclusively breast milk or appropriate formulas for about 6 months [8]. However, the infant’s needs increase after the sixth month, emphasizing the need for additional nutrients. The first 6–24 months is the transition period for the infant and child to complementary feeding [7]. According to the World Health Organization (WHO) and other organizations, differences exist regarding the appropriate time for transition to complementary feeding. WHO are implicit that exclusive breastfeeding to 6 months of age is optimal for most infants and that complementary feeding should start at about 6 months. According to the European Society for Paediatric Gastroenterology Hepatology and Nutrition (ESPGHAN), the transition to complementary feeding is recommended no earlier than the 17th week and no later than the 26th week. The main reasons for waiting until the sixth month for transition to complementary feeding are the maturation of the gastrointestinal system, strengthening of renal excretion, head control, and development of chewing movement [9, 10]. Complementary feeding is defined as giving foods other than breast milk, formula, medicines, vitamins, and mineral syrups to meet the nutritional needs of an infant from the sixth month onward when breastfeeding alone is no longer sufficient [11]. The complementary feeding period is a critical time of transition in infants characterized by a gradual shift from breast milk to family food [12].

A large part of brain development is completed between 0 and 2 years of age [13]. The rate of brain growth is such that the brain of a newborn infant will grow from 25% of its adult size to 80% by 24 months of age. Especially the time between 6 and 24 months is a period of surprisingly rapid neuronal interaction in the brains of infant and child. During this period, the regions necessary to control language, cognition, and emotions are actively developing [14]. For example, iron, which is among the micronutrients, is involved in the signal control of some neurotransmitters (dopamine and serotonin) in the nervous system [15]. In a review article, iron deficiency anemia before 24 months of age was found to increase the risk of poor cognitive, motor, social-emotional, language, behavioral, and neurophysiological development. It is also associated with a 6- to 15-point drop in developmental test scores compared with that in iron-sufficient infants and children, with deficits in cognitive and school achievement lasting into adolescence [16]. Therefore, the timing and amount of new food provided in the first 0–2 years of life can have both positive (high cognitive intelligence, vision, self-esteem, protection from noncommunicable diseases, increased growth rate, development of numerical and language skills, etc.) and negative (food allergy, hypoglycemia, fever, upper respiratory tract infection, anemia, growth retardation, asthma, diarrhea, etc.) effects in later periods. The timing of the introduction to new foods and their quality both affect nutritional patterns and the development of infants and children [13].

The ESPGHAN considers an early transition period to complementary feeding to be before 17 weeks (before 4 months) [9]. Early initiation of complementary feeding (before 4 months) may result in increased renal solute load and increased risk of obesity, malnutrition, infection, and allergy. On the contrary, delayed complementary feeding may result in slow growth, malnutrition, vitamin and mineral deficiency, difficulty adjusting to different tastes, and delayed chewing skills [9]. The age of initiation to complementary feeding may also influence the infant’s and child’s future food preferences and eating behavior [17]. Evidence shows that infants who transition to complementary feeding early (before 4 months) are more likely to consume more fatty or sugary foods at 1 year of age [18]. A comprehensive cohort study reported a reduced ability of infants and children receiving solid foods early in life to recognize satiety signals at the age of 5 years; however, late introduction to solid foods (after 7 months) was associated with reduced food enjoyment and food sensitivity [17].

Receiving complementary feeding is critical not only in terms of its impact on the rapidly increasing physical needs of infants and children but also in the formation of lifelong taste preferences and eating habits affecting long-term health [19]. Mealtimes are cultural and social events where infants and children observe and imitate their elders, develop their own likes and dislikes, and form lifelong eating habits and practices. Hence, paying attention to how and what infants and children are fed is important [13]. However, making mealtimes enjoyable is not always easy. Complementary feeding may be associated with various psychosocial challenges (stress, anxiety, feeling inadequate, lack of autonomy, social isolation, depression, etc.) for both mother and infant/child [20].

Meeting the infant’s and child’s nutritional needs and ensuring that the infant and child is fed enough can be stressful for mothers [20]. Maintaining a balance between feeding an infant and child and other responsibilities such as work, housework, and caring for other children can overwhelm mothers [21]. Mothers often take more responsibility than fathers while raising an infant and child, and an increase in roles and responsibilities can lead to different emotional changes. Providing mothers with support to cope with stress and anxiety and maintain their psychological well-being is essential [20].

Timely transition to complementary feeding is associated with various factors such as the socioeconomic status of the family, age of the mother, place of residence, educational level, number of infants/children, age of infants/children, and duration of breastfeeding [22]. Understanding the factors influencing complementary feeding is crucial to identifying practices supporting optimal complementary feeding [23]. Previous studies have focused on the factors influencing early or late transition to complementary feeding [22, 23]. A timely transition to complementary feeding facilitates the infant’s and child’s self-feeding in the early period and provides a faster transition to family meals [24]. During the complementary feeding period, the behaviors of infant and child, such as food reluctance, restlessness, and refusal of food, may affect the growth and development of the infant and child in the future [24, 25]. During this period, the behaviors of infant and child, such as unwillingness to eat, restlessness, and refusal of food, are defined as behaviors observed during the process of complementary feeding [25].

No study was found to examine the factors affecting the behaviors of 6- to 24-month-old infants and children during the process of complementary feeding. The findings of this study may provide a basis for future investigations in this area. Despite considerable evidence of the importance of the first 1000 days in determining long-term health outcomes, a significant gap exists in understanding the specific factors influencing complementary feeding behaviors during the critical 6- to 24-month period. This study aimed to investigate the social, cultural, and psychological factors that shape behaviors during the complementary feeding process, thus laying the groundwork for future research and interventions.

## Materials and methods

### Type of the study

The study was designed as a descriptive, predictive correlational, and cross-sectional study.

### Population and sample of the study

This study was performed on mothers with infants and children aged 6–24 months. The sample size for the study was calculated using the G*Power 3.1.9.7 program, specifically for multiple linear regression analysis. With a low effect size (*f*^2^ = 0.05), 5% margin of error (*α* = 0.05), and 80% power (1 − β = 0.80), the required sample size was determined to be 309. Considering possible data loss, the sample size was increased by 10%, and 330 participants were included in the study [26, 27]. Mothers with infants and children aged 6–24 months in the process of complementary feeding were included, whereas mothers of 6- to 24-month-old infants and children with health problems affecting their nutrition were excluded. This study was conducted in Turkey and data were collected online via social media. The use of social media allowed for the inclusion of participants from various regions of the country.

### Ethical considerations

This study was approved by the Social and Humanities Ethics Committee of Recep Tayyip Erdoğan University, with the decision numbered 2023/261 and dated 27.09.2023. It was conducted in line with the Helsinki Declaration. The ethics committee’s documentation included the following details regarding the data collection process and participant consent, and approval was granted based on these details:

- Research data were collected via Google Forms through social media channels such as Facebook and Instagram.
- The first part of the online data collection form consisted of an informed, voluntary consent form including details of the study and the contact information of the researchers. At the end of the consent form, participants could select “I want to participate in the research” or “I do not want to participate in the research.”
- Mothers who selected “I want to participate in the research” after reading the informed consent form were able to proceed to the second part, which contained the research questions.
- Mothers who selected “I do not want to participate in the research” after reading the informed consent form could not proceed to the second part containing the research questions, and the survey ended for them.

The permission to use the scale in the study was obtained via e-mail from the researcher who developed it.

### Data collection

The data were collected via social media channels (e.g., Facebook and Instagram) using a Google form and convenience and snowball sampling methods in Turkey. The online data collection form was shared on both the research team’s social media accounts and those followed by mothers with infants/children throughout the data collection process. Mothers with children aged 6–24 months were invited to participate in the study. The first part of the online data collection form included a voluntary consent form for mothers to read and approve online before participating in the study. The form also included the researchers’ contact information. Only the mothers who provided voluntary consent online proceeded to the second part with the research questions. The recruitment period for this study started on 06/10/2023 and ended on 08/01/2024. Eventually, 345 mothers who met the inclusion criteria and agreed to participate voluntarily were included in the study.

#### Data collection tools

Research data were collected using two forms and one scale. The Data Collection Form for Mothers and the Data Collection Form for Infants and Children were developed by researchers in line with the literature. The Behaviors of Transition to Complementary Feeding Scale (BTCFS) is a validated and reliable scale [25].

#### Data collection form for mothers

This form, developed by the researchers in line with the literature, included questions about the mother’s education, employment, income levels, presence of chronic disease, presence of support in childcare (e.g., from a caregiver, grandmother, or parental grandmother), and sources of information on complementary feeding (e.g., Internet pages, social media pages, books, health professionals, relatives, and friends) [22, 28, 29].

#### Data collection form for infants and children

This form was also developed by the researchers in line with the literature. Regarding the age of the infant/child, the cutoff point was taken as the 12th month (6–12 and 13–24 months), when the child started to feed himself/herself with hand coordination, especially the development of the kidney and gastrointestinal system, and started to participate in family meals in line with the sources [30, 31]. The form included birth weight (low: <2.5 kg, normal: 2.5–4 kg, and high: >4 kg) [32, 33], birth week (preterm: ≤37 weeks and term: ≥37 weeks) [33], sex (boy or girl), mode of delivery (cesarean section or vaginal delivery), type of nutrition for the first 6 months (breast milk–formula or breast milk only), current nutrition (breast milk–complementary feeding, breast milk–formula–complementary feeding, formula–complementary feeding, and only complementary feeding), and the presence of a food allergy diagnosed by a doctor and support from the father in infant/child care (low: 1–3, moderate: 4–8, and high: 8–10) [13, 29, 34].

#### Behaviors of transition to complementary feeding scale

The BTCFS was developed by Arslan et al. (2024) to determine the psychosocial behaviors experienced by infants and children aged 6 to 24 months during the process of complementary feeding [25]. The scale consists of 5 sub-dimensions, containing a total of 28 items. The items in this 5-point Likert-type scale are scored as follows: 1 = never, 2 = rarely, 3 = sometimes, 4 = often, and 5 = always. Items 2, 5, 9, 10, 11, 12, 13, 18, 19, 20, 21, 22, 23, 24, 25, and 26 are reverse-scored. The scores obtained from each subdimension (positive nutrition time, willingness to be fed, negative nutrition time, unwillingness to be fed, and refusal to be fed) are summed to obtain the total score; a high score indicates positive feeding behavior during the process of complementary feeding. The minimum and maximum scores on the scale are 28 and 140, respectively. Item-total correlations for this scale varied between 0.30 and 0.83 (p<0.001). The total explained variance was 64.56%. In exploratory factor analysis, the factor loadings for the scale ranged between 0.47 and 0.85. In confirmatory factor analysis, the factor loadings for the scale ranged between 0.46 and 0.92. Examples of the items are “My baby eats with an appetite,” “My baby likes feeding times,” “My baby looks happy while being fed,” “My baby spits food out,” and “My baby refuses new foods.” The Cronbach’s alpha value of the scale was found to be 0.95 in the scale development study.

### Statistical analysis

The data were analyzed using the Statistical Package for the Social Sciences Statistics (SPSS) 25.0 (IBM Corp., NY, USA). The mean, standard deviation, and percentile values were calculated for descriptive data. The conformity of the data to normal distribution was determined by calculating the kurtosis and skewness values. The kurtosis and skewness values in the range of +2 and –2 were taken as a reference for normal distribution [35]. Cronbach’s alpha coefficient was calculated to determine the internal consistency of the BTCFS. A Cronbach’s alpha coefficient of 0.70 and above was taken as a reference to ensure internal consistency [36]. Inferential statistics, which are among the analytical methods of quantitative research, were calculated using multiple linear regression analysis in line with the predictive correlational design. Before conducting the regression analysis, two assumptions of regression analysis—absence of autocorrelation and absence of multicollinearity—were examined. Autocorrelation was assessed using the Durbin-Watson statistic, while multicollinearity was evaluated by calculating the variance inflation factor VIF and tolerance values. The Durbin-Watson statistic measures the autocorrelation of errors across a sequence of cases. Positive autocorrelation makes estimates of error variance too small, resulting in an inflation of the Type I error rate. Negative autocorrelation makes these estimates too large, leading to a loss of statistical power. In the Durbin-Watson statistic, values between 1.5 and 2.5 generally indicate the absence of autocorrelation [37]. The Durbin–Watson test statistic value was 2.11, indicating no autocorrelation. When there are multiple predictor variables, relationships among the predictors can affect both the regression estimates and the standard error of the regression estimates. Multicollinearity occurs when any single independent variable is highly correlated with a set of other independent variables. In this case, one independent variable can be perfectly predicted by another independent variable (or by multiple others). In multicollinearity, one variable may be a combination of two or more other variables. Absence of multicollinearity is indicated when the tolerance is not less than 0.1 and the VIF does not exceed 10 [37]. As a result of the calculations, tolerance values were not less than 0.1 and VIF values did not exceed 10, indicating an absence of multicollinearity. The effects of the independent variables of infant’s and child’s age, birth weight, birth week, sex, rank among other children in the family, mode of birth, nutrition type in the first 6 months, current nutrition type, food allergy, and support from the father on the behaviors during the process of complementary feeding were examined using multiple linear regression analysis (Enter method). The significance level was set at *p* < 0.05.

## Results

The sociodemographic characteristics, childcare support, and information sources on complementary feeding for the mothers with infants and children on complementary feeding who participated in the study are presented in Table 1. The mean age of the mothers was 30.38 ± 3.72 years (min = 21, max = 48 years). Among these, 68.7% were university graduates, 57.7% were employed, and 51.3% had an income equal to their expenses. Further, 93.0% had no chronic disease, and 34.2% received support in childcare from a maternal grandmother, 26.4% from a paternal grandmother, and 10.1% from a caregiver. In addition, 64.6% received information about complementary feeding from Internet pages, 68.7% from social media pages, 47.2% from books on complementary feeding, 67.5% from healthcare professionals, and 46.7% from relatives and friends.

**Table 1:** Mothers’ sociodemographic characteristics, childcare support, and information sources on complementary feeding.

		*n*	%
**Education level**	High school	34	9.9
	Undergraduate	237	68.7
	Postgraduate	74	21.4
**Employment status**	Employed	199	57.7
	Unemployed	146	42.3
**Income status**	Income less than expenses	63	18.3
	Income equal to expenses	177	51.3
	Income more than expenses	105	30.4
**Presence of chronic disease**	Yes	24	7.0
	No	321	93.0
**Support from maternal grandmother in childcare**	Yes	118	34.2
	No	227	65.8
**Support from paternal grandmother in childcare**	Yes	91	26.4
	No	254	73.6
**Support from a caregiver in childcare**	Yes	35	10.1
	No	310	89.9
**Information source for complementary feeding = Internet pages**	Yes	223	64.6
	No	122	35.4
**Source of information for complementary feeding = Social media pages**	Yes	237	68.7
	No	108	31.3
**Source of information for complementary feeding = Books on complementary feeding**	Yes	163	47.2
	No	182	52.8
**Source of information for complementary feeding = Healthcare professionals (dietitian, nurse, midwife, physician, etc.)**	Yes	233	67.5
	No	112	32.5
**Source of information for complementary feeding = Relatives and friends**	Yes	161	46.7
	No	184	53.3

The sociodemographic characteristics, allergy presence, and feeding status of infants and children on complementary feeding are presented in Table 2. Of these infants and children, 49.6% were aged between 6 and 12 months, 49.6% were male, 82.9% were in the vaginal delivery birth range, and 81.7% were term. In addition, 71.9% were the first child, only one-third were term, 64.9% were exclusively breastfed for the first 6 months, 57.4% were breastfed and provided complementary feeding, and 12.5% experienced food allergies. Foods leading to allergies were milk, eggs, tomatoes, walnuts, and so forth.

**Table 2:** Sociodemographic characteristics, allergy presence, and feeding status of infants and children.

		*n*	%
**Month**	6–12	171	49.6
	>12	174	50.4
**Birth weight**	Low (<2.5 kg)	31	9.0
	Normal (2.51–4.0 kg)	286	82.9
	High (>4.0 kg)	28	8.1
**Birth week**	≤37 (preterm)	63	18.3
	≥37 (term)	282	81.7
**Sex**	Male	171	49.6
	Female	174	50.4
**Rank among other children**	1st	248	71.9
	2nd and above	97	28.1
**Mode of delivery**	Vaginal delivery	114	33.0
	Cesarean section	231	67.0
**Nutrition type for the first 6 months**	Breast milk + formula	121	35.1
	Breast milk only	224	64.9
**Current nutrition type**	Breast milk + complementary feeding	198	57.4
	Breast milk + formula + complementary feeding	36	10.4
	Formula + complementary feeding	61	17.7
	Complementary feeding only	50	14.5
**Presence of a food allergy diagnosed by a doctor**	Yes	43	12.5
	No	302	87.5
**Father’s support in childcare**	Low	39	11.3
	Moderate	142	41.2
	High	164	47.5

The mean total score of the BTCFS for the infants and children was 108.51 ± 16.53 (min = 41, max = 140). The kurtosis, skewness, and Cronbach’s alpha values were 1.17, –0.86, and 0.936, respectively. The mean total score of the BTCFS data met the normal distribution criteria based on skewness and kurtosis. The effects of characteristics of infants and children on the behaviors during the process of complementary feeding were examined using the multiple linear regression analysis (Enter method). The results of the analysis are shown in Table 3.

**Table 3:** Associations between infants’ and children’s characteristics and their behaviors during the process of complementary feeding.

Variable	*B*	*SE*	95% CI	β	*t*	*p*
(Constant)	116.20	2.62	(111.05–121.35)	0.00	44.38	<0.001
**Month (*R* = 6–12)**						
>12	–7.75	1.89	(–11.46 to –4.03)	–0.23	–4.10	**<0.001**
**Weight at birth (*R* = vaginal delivery)**						
Low	1.28	3.33	(–5.28 to 7.84)	0.02	0.38	0.702
High	–0.03	3.21	(–6.34 to 6.28)	–0.0004	–0.008	0.993
**Birth week (*R* =** ≥ **37)**						
≤ 37	0.21	2.50	(–4.71 to 5.12)	0.005	0.08	0.934
**Sex (*R* = female)**						
Male	–1.63	1.75	(–5.07 to 1.81)	–0.05	–0.93	0.351
**Rank (*R* = 2nd child)**						
1st child	–5.36	1.93	(-9.16 to –1.56)	–0.15	–2.78	**0.006**
**Mode of delivery (*R* = vaginal delivery)**						
Cesarean section	3.10	1.90	(–0.64 to 6.84)	0.09	1.63	0.104
**Nutrition in the first 6 months (*R* = breast milk only)**						
Breast milk + formula	–0.62	2.37	(–5.29 to 4.05)	–0.02	–0.26	0.794
**Current nutrition (*R* = breast milk + complementary feeding)**						
Breast milk + formula + complementary feeding	–3.80	3.36	(–10.42 to 2.81)	–0.07	–1.13	0.259
Formula + complementary feeding	–4.97	2.83	(–10.55 to 0.60)	–0.11	–1.75	0.080
Complementary feeding only	6.54	2.86	(0.91–12.16)	0.14	2.29	**0.023**
**Presence of a food allergy diagnosed by a doctor (*R* = no allergy)**						
Have an allergy	1.64	2.61	(–3.49 to 6.78)	0.03	0.63	0.529
**Father’s support (*R* = high)**						
Low father’s support	–7.00	2.90	(–12.71 to –1.29)	–0.13	–2.41	**0.016**
Moderate father’s support	–0.49	1.84	(–4.11 to 3.13)	–0.01	–0.27	0.789

Durbin–Watson = 2.21, *F* (14.330) = 3.08, *p* < 0.001, *R*^2^ = 0.12, and adjusted *R*^2^ = 0.08. CI: Confidence interval; SE: standard error; β: standardized regression coefficient. *Significance level (*p* < 0.05). Dependent variable = Behavior during the process of complementary feeding.

The model was statistically significant [*F*(14, 330) = 3.08, *p* < 0.001]. The variables that statistically significantly predicted behaviors during the process of complementary feeding in the model, in order of importance, were as follows: (1) Age of the infant/child (β = –0.23, *p* < 0.001): Infants and children aged >12 months were found to have more negative behaviors during the process of complementary feeding compared with those aged 6–12 months. (2) Rank of the infant among other children in the family (β = –0.15, *p* < 0.006): This study showed that being the first infant/child in the family negatively affected behaviors during the process of complementary feeding. (3) Current diet of the infant/child (β = 0.14, *p* < 0.023). The combination of complementary feeding and breast milk negatively predicted the behaviors during the process of complementary feeding. (4) Support from the father in childcare (β = 0.13, *p* < 0.016): Father’s support in childcare positively predicted the behaviors during the process of complementary feeding.

## Discussion

Nutrition is a process that begins in the intrauterine period and continues throughout life. Breast milk is an important food source providing adequate nutrition and protection against infections in the first 6 months of life [38]. Numerous guidelines state that infants should be exclusively breastfed for the first 6 months [10, 39]. However, breast milk alone is insufficient to meet the infant’s nutritional needs after the first 6 months [40]. Therefore, transitioning to complementary feeding to meet the nutritional needs of infants is essential. Complementary feeding is defined as “food and fluid consumption when breast milk or follow-on formula is no longer sufficient to meet the needs of infants” [9]. The complementary feeding period covers the first 6th month to the 24th month. This is a sensitive period during which the infant and child are exposed to new foods, tastes, and experiences besides breast milk, and dietary habits are formed [41].

Psychosocial problems experienced by infants and children provided complementary feeding for the first time influence their nutritional patterns in the future. The infant’s and child’s nutritional pattern and behavior developed during this period can be crucial for the future development of the infant and child [5, 42]. Erroneous practices during the process of complementary feeding, such as forcing the infant and child to eat or consume food quickly, may lead to the adoption of unhealthy eating habits in the future [43]. This eventually results in issues in the growth and development of the infant/child and noncommunicable diseases such as obesity, diabetes, and heart disease. Hence, the complementary feeding period has gained increasing interest from researchers [5]. This interest has been effective in addressing the factors influencing complementary feeding. The present study addressed the factors affecting behaviors during the complementary feeding process.

The most important factor influencing behaviors during the process of complementary feeding was found to be the age of the infant and child. Infants and children aged > 12 months were found to have more negative behaviors during the process of complementary feeding compared with those aged 6–12 months. The physical development of infants and children covers different periods such as sitting (6–8 months), crawling (8–12 months), and toddling (12–15 months) [44]. Mothers are responsible for feeding infants in the sitting and crawling periods (the first 6–12 months) [45]. However, the mother’s role in feeding decreases in the toddler period [44]. The rapid change in the mood of toddlers, their choices of food, refusal to eat, and unwillingness to eat new foods make the process of complementary feeding difficult. Infants/Children aged >12 months, who are keen to explore new things and become independent with walking, can perceive everything as play, which can cause variation in their complementary feeding behaviors [2, 29]. In the present study, infants/children aged >12 months had more unfavorable behaviors during the complementary feeding process. This was probably due to the desire of infants and children to explore and form a sense of self. Food choice can be supported to improve the independence and sense of self among infants and children during complementary feeding. In addition, promoting the exploring tendency of infants and making complementary feeding a fun presentation and activity for them can facilitate their process of complementary feeding in the toddler period.

The rank of infants/children among other children in the family is the second most important factor affecting behaviors during the complementary feeding process. This study showed that being the first infant/child in the family negatively affected the behaviors during the complementary feeding process. One important issue in the formation of the food preferences of infant and child is the presence of older siblings at home, which significantly influences the infant’s and child’s nutrition and food preferences, especially during the preschool period [46]. In addition, the mother’s previous experience with complementary feeding increases her self-confidence and reduces complementary feeding-related anxiety [47]. Therefore, infants and children with older siblings during the complementary feeding period have more positive behaviors during the complementary feeding process. This may be because the infant and child may consider the older sibling as a role model during this period. Moreover, it is thought that mothers with complementary feeding experience may have less anxiety about complementary feeding, increasing their confidence during the complementary feeding process. The anxieties of mothers whose first infant/child is in complementary feeding process should be understood, and training should be provided to reduce their anxiety. Mothers of second infants/children, who have experienced difficulties in introducing complementary feeding to their first infant/child, can gain more information about complementary feeding from healthcare professionals or other reliable sources to help, at this stage, with their second infant/child.

The third important factor affecting the behavior of infants and children during complementary feeding is the current nutritional pattern. In this study, it was determined that the behaviors of infants and children who received only complementary feeding were more positive than those who received both breast milk and complementary feeding. This may be due to the infant’s and child’s late transition to complementary feeding or the implementation of a feeding plan with an emphasis on breast milk during the complementary feeding period. Breast milk is essential for the growth of infants and children. The analysis of the content of breast milk shows that breast milk is sufficient to meet the nutritional needs of most infants in the first 6 months. However, after the sixth month, breast milk cannot meet the needs of the infant and child in terms of average energy, protein, and some micronutrients (such as iron) [22]. Breastfeeding is a protective factor for the good development of oral muscles, chewing, sucking, and general development of the oral cavity of infant and child. It is also important for establishing and developing an emotional connection between the infant/child and the mother [48]. However, consuming only breast milk for a long time may delay the transition to complementary feeding, leading to developmental and cognitive disorders and malnutrition (e.g., iron deficiency). Late transition to complementary feeding may make it difficult for the infant and child to accept the taste and texture of foods. It may also cause food refusal [22]. During the period to complementary feeding, the mother may have delayed the transition to complementary feeding by breastfeeding instead of offering additional food to the infant and child. The mother may not know the time when the infants and children shows signs of readiness in the transition to complementary feeding complementary feeding. At the same time, the mother’s misinformation about the complementary feeding period (social media, etc.) may have caused her behavior to be negative during the complementary feeding process. The importance of complementary feeding should be explained to mothers with infants aged 6 months. Also, they should be informed about the problems they may experience in case of delay.

Another factor influencing the infant’s and child’s behavior during the complementary feeding process is the father’s support in childcare. The present study found that infants and children whose fathers supported the mother in childcare had more positive behaviors during the complementary feeding process. Fathers can play essential roles during the complementary feeding process, besides their involvement in the development of the infant and child [49]. These roles include supporting the mother in childcare, deciding on a uniform feeding schedule for the infant and child, and assisting the mother in preparing and providing the food in complementary feeding.

Studies have shown that the father’s behaviors and attitudes, especially in the first 1000 days of the infant and child’s life, have major impacts on the their growth and development [34, 49]. The indifference and insensitivity of fathers to the development of the infant and child can negatively influence maternal behaviors. On the contrary, fathers’ support, especially in the first years of the infant’s and child’s life, can reduce psychological stress in mothers [34]. Thus, the father’s support for the mother’s psychological well-being positively influences the infant and child during the complementary feeding process [50]. Recent studies have also demonstrated a significant positive correlation between an increase in fathers’ support in childcare and infants’ nutrition [51, 52], consistent with the findings of this study. It should be emphasized that complementary feeding is the responsibility of both mothers and fathers. Parents should visit healthcare institutions, and fathers should be informed about their roles in complementary feeding. Fathers should be trained about the importance of meals, food presentation, and hygiene for the infant and child during the complementary feeding period. The father’s support for the mother during childcare can positively influence the mother–father–infant/child relationship.

The relationship between mother and infant/child is one of the critical factors regulating the complementary feeding period of infants and children. The mother’s emotional state during complementary feeding modulates the infant’s and child’s adaptation to complementary feeding [53]. An inconsistent maternal interaction can have a negative effect on the emotional development of the infant and child in complementary feeding. A mother who is anxious during complementary feeding should be informed about nutrition [54]. Mothers see healthcare professionals as the most important source of information on complementary feeding; however, they also frequently receive help from their relatives and the Internet [55]. A study on the information sources of mothers about the complementary feeding revealed that 76.7% of mothers received information from healthcare professionals, 19.9% from social media, and 36.5% from websites [56]. In the present study, 67.5% of the participants received information about complementary feeding from healthcare professionals, 68.7% from social media, and 64.6% from websites. This showed that the mother received much information about complementary feeding from the Internet and social media. Therefore, it is believed that the Internet may play an important role as a source of information about the complementary feeding period in parallel with the increase in Internet use. Two recent studies emphasized the existence of a lot of information about complementary feeding on social media and the Internet; however, the accuracy of this information was questionable [57, 58].

Supporting the mother psychosocially during complementary feeding process of infant and child positively influences complementary feeding behaviors [44]. The mother’s request for information and support from her relatives during the process of complementary feeding helps regulate her emotional state. A study reported that mothers needed support and information on complementary feeding from maternal grandmother, paternal grandmother, and caregiver [55]. The present study found that 34.2% of the mothers received support from the maternal grandmother, 26.4% from the paternal grandmother, and 10.1% from the caregiver. These data indicated that a large number of mothers needed someone’s support in childcare.

According to the Turkish Demographic and Health Surveys (TDHS) 2018 data, 98% of children born in the last 2 years in Turkey were breastfed for any period of time at any time [59]. A study conducted in Turkey in 2024 showed that the rate of infant who received no breast milk was 3.5% [60]. Each of the mothers who participated in this study breastfed their infants at any time during the first 6 months for any period of time. It is thought that this increase in the rate of breastfeeding is due to the awareness raised about the importance of breast milk.

**Fig 1:**
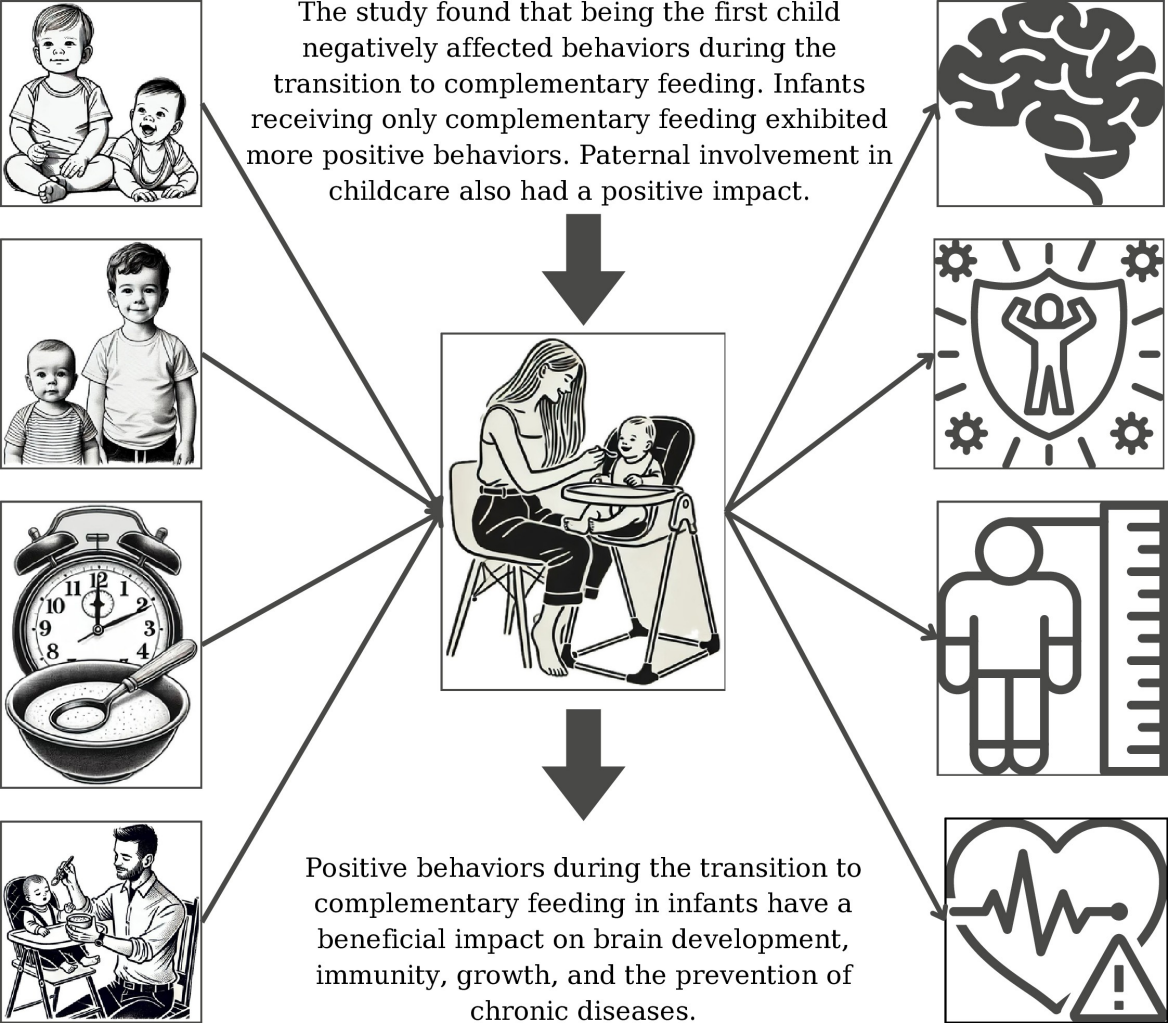
Factors affecting the behaviors during the complementary feeding process in infants and children and the effects of this process.

The factors influencing behaviors during the complementary feeding process in infants and children, and the effects of this process, are listed in Fig 1.

### Limitations

This study had some limitations. First, the data were collected using an online data collection form, and hence the possibility of bias cannot be ignored. The research findings were based on mothers’ self-reported responses to the survey questions. The mothers participating in the study had smartphones and Internet access to fill out the online data collection form, and their education and income levels were above average in Turkey. Therefore, the results cannot be generalized. Future studies should examine the behaviors during the complementary feeding process using larger sample groups including different settlements such as villages.

## Conclusions

The findings of this study revealed that age, rank among siblings, current nutritional pattern, father’s support, and the mother–infant/child relationship significantly influenced complementary feeding behaviors. The process of complementary feeding is complex, shaped by numerous factors such as the infant’s and child’s age, family interactions, and availability of parental support. We should offer tailored guidance to mothers, engage fathers actively, and provide accurate information to ensure a more effective complementary feeding process. These measures can help foster healthier feeding practices in infants and children. Healthcare professionals should consider the aforementioned factors when developing methods to support the complementary feeding process. Implementation of adequate strategies during this period can positively affect the growth and health status of infants and children in later life. In addition, parents should be informed about the factors influencing the behaviors during the complementary feeding process and the impact of these factors on the future life of infants and children. Descriptive, interventional, qualitative, and mixed-method studies should be conducted to analyze these factors in detail and improve the complementary feeding process.

## Supporting information

S1 Table Behaviors of transition to complementary feeding scale. (PDF)
