# Supplementary material for: Factors affecting behaviors during complementary feeding in infants and children aged 6–24 months
Source: PLoS One. 2025 Jan 3;20(1):e0314694. doi: 10.1371/journal.pone.0314694 (PMC11698433; doi:10.1371/journal.pone.0314694)
Supplement: S1 Table — (PDF) [file pone.0314694.s001.pdf]

Table 1. Behaviors of Transition to Complementary Feeding Scale

		never	rarely	sometimes	often	always
1	My baby enjoys feeding him/herself.					
2	My baby cries when feeding/eating					
3	My baby has fun feeding/eating					
4	My baby enjoys eating					
5	My baby needs music/ cartoons/ videos/ games when feeding/eating					
6	My baby feeds/eats with an appetite					
7	My baby likes mealtimes					
8	My baby looks happy when feeding/eating					
9	My baby is restless when feeding/eating					
10	My baby gets angry when feeding/eating					
11	My baby hurls or throws food on the floor at mealtimes					
12	My baby cries at feeding/mealtimes					
13	My baby is unwilling to feed/eat					
14	My baby is eager to feed him/herself					
15	My baby wants to taste everything that is put before him/her					
16	My baby picks up the food placed before him/her and puts it into his/her mouth					
17	My baby likes tasting new foods					
18	My baby rejects new food					
19	My baby keeps his/her food in his/her mouth for a long time					
20	My baby shuts his/her mouth at feeding/mealtimes					
21	My baby backs away when food is put before him/her					
22	My baby plays with his/her food when it is put in front of him/her					
23	My baby spits out his/her food					
24	My baby kicks, scratches or displays other aggressive behavior during feeding/mealtimes					
25	My baby rejects any new food put before him/her					
26	My baby does not like to eat food that smells different or is of a different consistency					
27	My baby enjoys his/her food					
28	My baby is calm between feedings/meals					

REFERENCE

Arslan N, Kürtüncü M, Turhan P.M. Behaviors of Transition to Complementary Feeding Scale (BTCF-S): A scale development study. Cyprus J Med Sci. 2024;9(3):192-197
